# The SPECTRUM Consortium: a new UK Prevention Research Partnership consortium focussed on the commercial determinants of health, the prevention of non-communicable diseases, and the reduction of health inequalities

**DOI:** 10.12688/wellcomeopenres.16318.1

**Published:** 2021-01-14

**Authors:** Marie Horton, Parvati R. Perman-Howe, Colin Angus, Julie Bishop, Ilze Bogdanovica, Alan Brennan, John Britton, Leonie S. Brose, Jamie Brown, Jeff Collin, Martin Dockrell, Niamh Fitzgerald, Sharon Friel, Duncan Gillespie, Anna B. Gilmore, Sarah E. Hill, Cecile Knai, Tessa Langley, Sancha Martin, Ann McNeill, Graham Moore, Marcus R. Munafò, Rachael L. Murray, Magdelena Opazo Breton, Jamie Pearce, Mark Petticrew, Garth Reid, Deborah Robson, Harry Rutter, Lion Shahab, Niamh Shortt, Katherine Smith, Keith Syrett, Linda Bauld

**Affiliations:** 1 Population Health Analysis, Health Intelligence, Public Health England, London, UK; 2 Addictions Department, Institute of Psychiatry, Psychology and Neuroscience, King’s College London, London, UK; 3 School of Health and Related Research, University of Sheffield, Sheffield, UK; 4 Health Improvement Division, Public Health Wales, Cardiff, UK; 5 UK Centre for Tobacco and Alcohol Studies, Division of Epidemiology and Public Health, Faculty of Medicine and Health Sciences, University of Nottingham, Nottingham, UK; 6 Department of Behavioural Science and Health, UCL, London, UK; 7 Global Health Policy Unit, Social Policy, School of Social and Political Science, University of Edinburgh, Edinburgh, UK; 8 Health Improvement Directorate, Public Health England, London, UK; 9 Institute for Social Marketing & Health, University of Stirling, Stirling, UK; 10 Centre for Health Governance, School of Regulation and Global Governance, Australian National University, Canberra, Australia; 11 Tobacco Control Research Group, Department for Health, University of Bath, Bath, UK; 12 Faculty of Public Health and Policy, London School of Hygiene and Tropical Medicine, London, UK; 13 Usher Institute, College of Medicine and Veterinary Medicine, University of Edinburgh, Edinburgh, UK; 14 DECIPHer, School of Social Sciences, Cardiff University, Cardiff, UK; 15 School of Psychological Science and MRC Integrative Epidemiology Unit, University of Bristol, Bristol, UK; 16 Centre for Research on Environment, Society & Health, University of Edinburgh, Edinburgh, UK; 17 Public Health Science Directorate, Public Health Scotland, Edinburgh, UK; 18 Department of Social and Policy Sciences, University of Bath, Bath, UK; 19 School of Social Work & Social Policy, University of Strathclyde, Glasgow, UK; 20 Centre for Health, Law, and Society, School of Law, University of Bristol, Bristol, UK; 21 Alcohol, Drugs, Tobacco and Justice Division, Health Improvement Directorate, Public Health England, London, UK

**Keywords:** Public health, Policy, Inequalities, Non-communicable disease, Prevention, Tobacco, Alcohol, Unhealthy commodities, Commercial determinants

## Abstract

The main causes of non-communicable diseases (NCDs), health inequalities and health inequity include consumption of unhealthy commodities such as tobacco, alcohol and/or foods high in fat, salt and/or sugar. These exposures are preventable, but the commodities involved are highly profitable. The economic interests of ‘Unhealthy Commodity Producers’ (UCPs) often conflict with health goals but their role in determining health has received insufficient attention. In order to address this gap, a new research consortium has been established. This open letter introduces the SPECTRUM ( **S** haping **P**ublic h **E**alth poli **C**ies **T**o **R**educe ineq **U**alities and har **M)**Consortium: a multi-disciplinary group comprising researchers from 10 United Kingdom (UK) universities and overseas, and partner organisations including three national public health agencies in Great Britain (GB), five multi-agency alliances and two companies providing data and analytic support. Through eight integrated work packages, the Consortium seeks to provide an understanding of the nature of the complex systems underlying the consumption of unhealthy commodities, the role of UCPs in shaping these systems and influencing health and policy, the role of systems-level interventions, and the effectiveness of existing and emerging policies. Co-production is central to the Consortium’s approach to advance research and achieve meaningful impact and we will involve the public in the design and delivery of our research. We will also establish and sustain mutually beneficial relationships with policy makers, alongside our partners, to increase the visibility, credibility and impact of our evidence. The Consortium’s ultimate aim is to achieve meaningful health benefits for the UK population by reducing harm and inequalities from the consumption of unhealthy commodities over the next five years and beyond.

## Background

The main causes of non-communicable diseases (NCDs) include consumption of unhealthy commodities such as tobacco, alcohol or foods high in fat, salt and/or sugar. These exposures are preventable, but the commodities involved are highly profitable. Their consumption and health and social impacts are also inequitable and driven by complex systems of production, distribution and promotion dominated by transnational companies - Unhealthy Commodity Producers (UCPs). UCPs’ economic interests often conflict with health goals and their role in determining health has received insufficient attention.

SPECTRUM (Shaping Public hEalth poliCies To Reduce ineqUalities and harM) is a multi-university, multi-agency research consortium that aims to shape public health policies and practices that focus on the prevention of NCDs and the reduction of health inequalities caused by the consumption of unhealthy commodities ^1^. It is funded by the UK Prevention Research Partnership (UKPRP) ^2^, and builds on the strong foundation of the UK Centre for Tobacco and Alcohol Studies (UKCTAS): a strategic partnership funded by the UK Clinical Research Collaboration that included 14 university academic groups working collaboratively on research, teaching, training and policy development to prevent harms to health and wider society arising from tobacco and alcohol use between 2013 and 2018.

SPECTRUM brings together researchers from 10 UK Universities with support from academics overseas. Our work will be developed with a range of partner organisations including health bodies, charities, and companies who provide data and whose staff have skills that can enhance our research. We will involve the public through regular meetings with groups made up of members of the public (user panels), specific meetings such as citizens’ juries, and by interviewing people from communities across the country. We will work with our partners, politicians, civil servants, and health and social care professionals amongst others to jointly design and deliver research so that it meets public and professional needs. We anticipate SPECTRUM will accelerate and improve the use of accelerate the use of evidence to inform new and existing health policy and practices in the UK, and to develop research that will also support progress in other countries.

### Priority areas

SPECTRUM will generate new evidence to inform the prevention of NCDs caused by unhealthy commodities focusing on tobacco and alcohol and now extending work to unhealthy food and drinks. 

***Tobacco.*** Smoking is the leading cause of preventable illness and premature mortality in the UK and around the world ^3^. It is a major risk factor for many diseases including lung cancer, chronic obstructive pulmonary disease (COPD), stroke and heart disease ^4^. Smoking is responsible for an estimated 95,000 deaths per year in the UK and costs society approximately £12.5 billion per year in England ^5, 6^.

There has been a significant decline in the proportion of smokers in the UK in recent years: from 20.2% in 2011 to 14.1% in 2019 ^6^. However, this still equivalent to an estimated 6.9 million smokers in the UK in 2019 ^6^. There are large inequalities in the proportion of current smokers by deprivation, ethnic group, sexuality, occupation, mental health status and geographical area of residence ^4^. These inequalities exist despite robust evidence of effective tobacco control measures that can reduce smoking prevalence at the population level.

***Alcohol.*** Alcohol consumption is a causal factor in more than 200 physical and mental health conditions and intentional and non-intentional injuries ^7, 8^. Alcohol misuse is the fifth biggest risk factor for preventable illness, premature mortality, and disability in England, for example, and the leading risk factor amongst those aged 15 to 49 ^9^. In 2018, there were 7,551 avoidable deaths in the UK that were caused by alcohol ^10^.

Alcohol-related harm disproportionately affects those with low individual or neighbourhood socioeconomic status/position (SES) ^11– 13^. Those in low SES classifications are at almost twice the risk of alcohol-related mortality than individuals in higher SES classifications ^11^.

It is estimated that the total cost of alcohol-related harm was 2.5% of gross domestic product (GDP) of high-income countries in 2007: equivalent to £47 billion in the UK in 2016 ^14, 15^.

***Unhealthy food and drinks.*** Almost two thirds of adults in the UK are overweight or obese, and in 2019 more than one in three children in England left primary school overweight or obese ^16, 17^. Excess weight is a leading cause of diabetes, cardiovascular disease, at least 12 different types of cancer, and mental ill-health ^18, 19^. Obesity-related illnesses cost the NHS around £6 billion per year ^20^.

Obesity prevalence is greater with higher levels of deprivation, and children living in the most deprived parts of the country are more than twice as likely to be obese as children living in the least deprived areas ^17^.

Unhealthy diets that include food and drinks that are high in fat, sugar and/or salt (HFSS) are a major contributor to overweight and obesity. In July 2020, the Government published a new obesity strategy that, when implemented, will aim to change elements of the food environment ^21^. The strategy proposes a future ban on advertising HFSS products online and on TV before 9pm, a restriction on promotional deals (such as ‘buy one get one free’) on HFSS products, and the requirement for food businesses with over 250 employees to provide calorie labels on food items ^21^.

## Aims and objectives

### Priority objectives

SPECTRUM’s five priority objectives are to:

1. develop and evaluate interventions to prevent harm from tobacco and alcohol use, and extend this work to HFSS food and drinks;
2. provide timely evidence to inform policy and practice;
3. understand and address industry strategies;
4. identify how systems can meet the challenge of these preventable risk factors;
5. understand the economic costs and benefits of system change and their implications for inequalities.

### Theory of change

**Figure 1.**
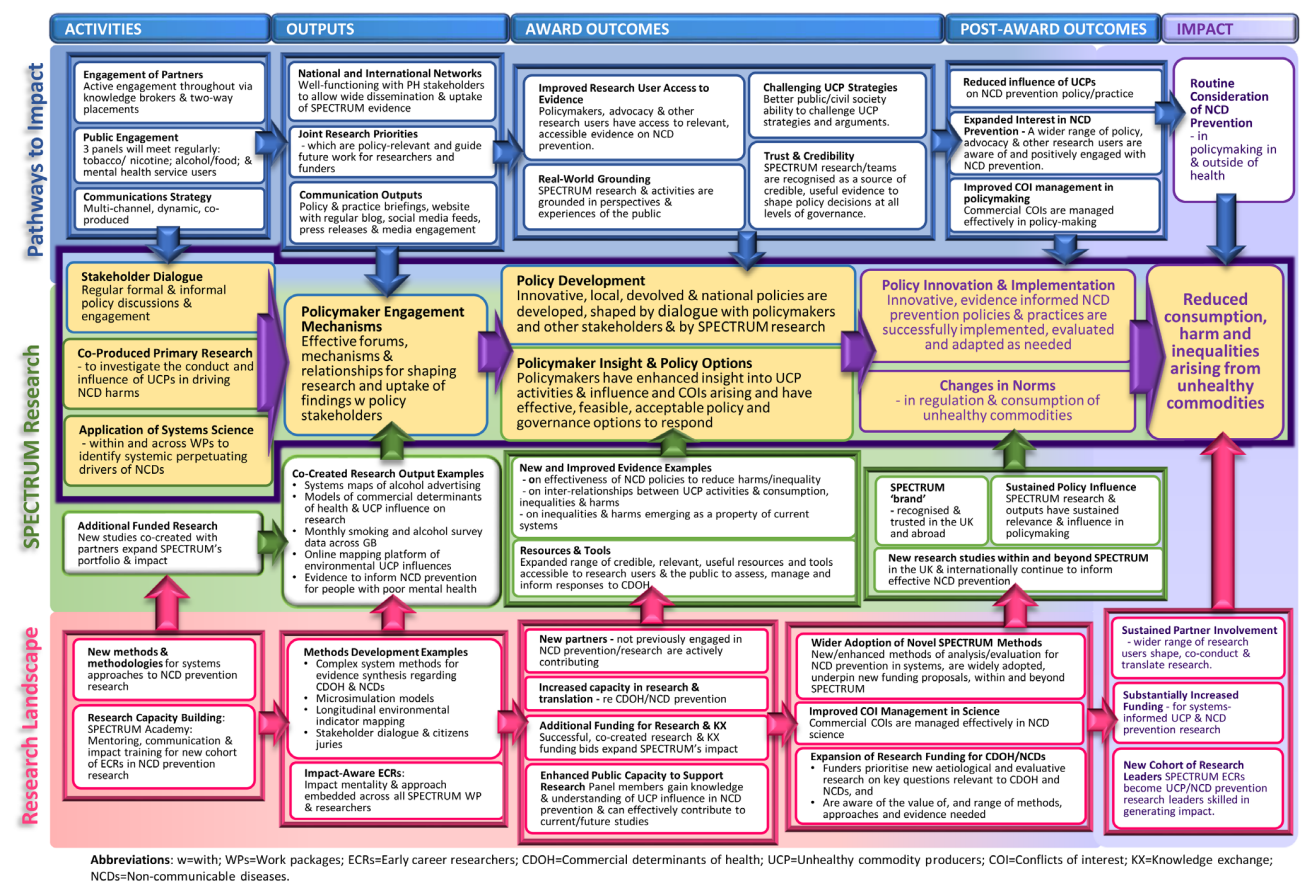
Theory of Change.

As illustrated in our Theory of Change ( Figure 1), we expect SPECTRUM to achieve meaningful benefits for the UK population, particularly by contributing to reduced consumption, harm and inequalities arising from unhealthy commodities in the medium to long term (5 to 10 years). This will be achieved by ensuring that we meet our impact goals which are described in more detail below (see *Impact and anticipated outputs*). The three main goals are:

1. co-produce evidence to underpin the development, enactment, evaluation and maintenance of large-scale generalizable and cost-effective interventions to reduce consumption, harm and inequalities arising from unhealthy commodities;
2. establish, enhance and sustain mutually beneficial relationships with policy and other partners to increase the visibility, credibility and impact of SPECTRUM evidence, and to make the case for future investment in NCD prevention and commercial determinants of health research;
3. embed sustainable and meaningful strategies for public involvement to ensure our research, outputs and activities are accessible and grounded in lived experience.

In the short to medium term, SPECTRUM research will support devolved and national governments to develop, enact and sustain evidence-informed, innovative policy and practice changes and strategies to reduce NCD harm and inequalities. For example, both the UK and Scottish Governments are considering new or revised alcohol, and tobacco strategies, including greater regulation of price, availability and promotions. Through SPECTRUM, researchers, advocacy organisations, non-governmental organisations (NGOs) and the public will co-create evidence to highlight and counter the influence of UCPs and develop cross-sectoral governance approaches. Working in coalition with advocacy and civil society partners, SPECTRUM will draw and build on existing relationships with policymakers to shape research to policy needs. Emerging evidence will be fed into a two-way dialogue leading to practice and policy change in local authorities, the NHS, devolved administrations and national government.

### SPECTRUM work packages (WPs)

SPECTRUM has eight integrated work packages, which together form a cohesive programme of work to inform our theory of change. The interactions between these will provide a layered understanding of the nature of the complex systems underlying the consumption of unhealthy commodities, the role of UCPs in shaping these and influencing health and policy, the role of systems-level interventions, and the effectiveness of existing and emerging policies.

***WP1: Using systems approaches to understand determinants and address harms***

WP1 aims to develop systems-based approaches and perspectives that can be applied to a range of UCPs and different types of research including new primary studies and evidence syntheses and then apply these in new case studies, chosen to allow the analysis of the system-level drivers of specific public health harms related to unhealthy commodities. WP1 will assess the potential for transferring a new conceptual model and general methodological approach to other unhealthy commodities or services (such as HFSS foods), to public-private partnerships, and to particular settings and/or populations (e.g. schools, and children and young people) within the whole system. WP1 aims to use a causal loop diagram approach to identify parts of the system within which there is potential for intervention, and to identify how under-researched sub-systems may facilitate and/or reduce the effectiveness of intervention.

***WP2: Understanding and addressing unhealthy commodity industry influence***

WP2 aims to understand how UCPs influence health and public policy, through targeted case studies of corporate political activity around emerging NCD policies, and develop a conceptual model for understanding the commercial determinants of health, with an emphasis on UCPs. WP2 will compile evidence of best practice through reviews of the academic and grey literature and key informant interviews (with creators and users of the research) aiming to identify the specific types of research and knowledge translation most likely to enable policy change. WP2 will develop a model of the commercial determinants of health, drawing on evidence of the diverse ways UCPs’ practices impact on health, the emerging body of literature profiling the commercial determinants of health, and the ongoing review of strengths and weaknesses of existing models of determinants of health.

***WP3: Developing and integrating new data sources to inform action***

The Smoking and Alcohol Toolkit Study (STS/ATS) ( www.smokinginengland.info and www.alcoholinengland.info) is a national monthly survey established in 2006 that aims to provide insight into population-wide influences on smoking and alcohol in England. Each month a new sample of around 1700 adults (age 16+ years) complete a computer-assisted household survey with a trained Ipsos MORI interviewer with around 270,000 responses recorded to date. Respondents are selected by a hybrid method between random location and simple quota sampling and the sample is nationally representative in its socio-demographic composition. WP3 will expand detailed monthly surveillance of preventable risk factors in the STS/ATS across GB and constituent nations using new participatory methods to refine data collection in response to evolving policymaker needs and public interest. WP3 will also work with WPs 4, 6 and 7 to develop and apply new methods from applied statistics and economics for integrating data and forecasting new trends and develop a microsimulation model of smoking and quitting in GB to identify emergent effects from divergent policy scenarios. 

***WP4: Economic and health impact evidence synthesis to inform policy and practice decisions***

WP4 will develop new evidence, modelling and analysis on the impacts of alcohol and tobacco, their associated diseases, and prevention policies on the wider economy, focussing on wider complex system effects of unhealthy commodities and prevention policies in the UK. WP4 will also develop and extend the Sheffield Tobacco and Alcohol Policy Model (STAPM) for England to analyse the economic and health impacts of changes in use of alcohol and/or tobacco on a wide range of outcomes and produce new versions for both Wales and Scotland, thus enabling between-country comparisons. WP4 will integrate this modelling and economic analysis with evidence from the other WPs to undertake policy evaluation (retrospective), appraisal (prospective), and scenario analyses (modelling) from an economic perspective, with a focus on the impact of UCP activities intended to defeat or delay regulation or policy implementation.

***WP5: Shaping the local environment to change behaviour and prevent harm***

WP5 aims to examine the intended and unintended impacts of (and interventions in) the local commercial environment on the consumption of unhealthy commodities by compiling a comprehensive interactive mapping platform to integrate data on alcohol, tobacco and food outlet and advertising locations with information tracking people’s mobility. To do this, WP5 will develop novel geographical analyses to model the impacts and unintended consequences of policy approaches designed to change local environments. WP5 will merge data on availability, price, marketing and norms to examine how these factors interact, building on the existing publicly accessible platform for mapping tobacco and alcohol environments in Scotland to develop a comparable resource for tobacco, alcohol and food retailing across GB.

***WP6: Evaluating the effectiveness of policies and natural experiments***

WP6 will assess the effects of tobacco and alcohol policies (such as standardised packaging for tobacco and MUP for alcohol in Scotland) on product diversity, price and consumption using a standardised framework, and extend the framework to food policy. WP6 will identify strategies used to circumvent advertising restrictions and potential inadequacies of existing regulatory approaches related to unhealthy commodity imagery in the media, and evaluate implementation of tobacco and alcohol treatment in the healthcare system.

***WP7: Disrupting the relationship between mental health, stigma and unhealthy commodities***

WP7 aims to understand the relation between unhealthy commodities, mental ill-health, and stigma, and to explore how these relationships can be disrupted over time by investigating the relationship between unhealthy commodities and mental ill-health, and the impact of UCPs, governmental and non-governmental initiatives on these relationships. Through the establishment of a new panel of people with mental health problems, WP7 will explore how people with mental ill-health who smoke and/or use alcohol perceive government mass media campaigns and UCP marketing campaigns and how these affect their behaviour and influence norms/stigmatization and evaluate new co-designed health communication strategies. WP7 will also examine how UCPs position their products in relation to social norms and mental health.

***WP8: Towards governance for health equity***

WP8 aims to identify how health governance and regulatory mechanisms can be used to develop coherent approaches to tackling the health and social impacts of unhealthy commodity industries. By understanding opportunities for and barriers to integrated approaches to NCD prevention, identifying potential mechanisms to promote co-ordination across policy areas that are typically siloed, WP8 will assess the adequacy of current practices via multiple methods including systematically reviewing researchers’ perceptions of conflict of interest and of the effectiveness of existing governance tools. WP8 will use citizens’ juries to build on work undertaken in WP5 to explore neighbourhood social norms and maximize the legitimacy and acceptability of policy innovation and of legal and other regulatory frameworks for NCD prevention.

## Plans for co-production and knowledge exchange

### SPECTRUM members and partners

The SPECTRUM Consortium includes 11 universities: Bath, Bristol, Cardiff, Edinburgh, Nottingham, Sheffield, Stirling, King's College London, London School of Hygiene and Tropical Medicine, University College London and the Australian National University. It also includes the main public health agencies (Public Health England, Public Health Scotland, and Public Health Wales) in Great Britain. Additionally, partners include The Retail Data Partnership, Sandtable, and the Alcohol Health Alliance, Smokefree Action Coalition, Obesity Health Alliance, NCD Alliance and the Poverty Alliance. Co-production is central to our approach to advancing research and achieving impact. Impact plans have been developed based on policy and advocacy needs identified through stakeholder engagement prior to and throughout the consortium development grant phase.

***Knowledge exchange.*** Our approach to knowledge exchange involves planned activities for the Consortium and each WP but will also be dynamic and flexible, building on existing strong relationships with research users. This flexibility is essential to feed evidence and co-produced intervention options arising from our research into policy debates at national and local level in a timely manner.

SPECTRUM will host events for stakeholders, bringing together policy makers, NGOs, public health and health professional bodies, lay representatives, co-applicants and partners, to share research findings and seek input on next steps. WPs will attend stakeholder and policy conferences, sharing their research through presentations and workshops to expand coalitions and engage new partners. SPECTRUM will deliver continuing professional development (CPD) workshops for academics, policy, and advocacy and practice colleagues on alcohol policy and tobacco/nicotine. These will be delivered by academics and partners and will facilitate knowledge exchange, capacity building, networking, policy influence, coalition building and bid development.

***Knowledge brokers.*** Three knowledge broker positions have been created as secondment posts from PHE, PHW and NHS Health Scotland. The main purpose of these roles is to facilitate co-production between the SPECTRUM WPs and public health agencies, build relationships with policymakers to support SPECTRUM to deliver high-impact, policy-relevant outputs, and to ensure researchers are front and central in live policy discussions. 

***Public involvement.*** We will work closely with the public through involvement panels, citizens’ juries and qualitative research. Three public involvement panels have been set up and will meet regularly to co-produce research, inform debate and shape the research agenda:

1. Alcohol (and Food Policy) Discussion Group.
2. Tobacco and Nicotine Discussion Group (TANG).
3. Mental Health Service Users’ Discussion Group.

## Impact and anticipated outputs

An effective communications strategy will be essential to achieving our three impact goals referred to previously

Our communications activities will be embedded within, and complementary to, collaborative working and ongoing two-way dialogue with research users, rather than being limited to publishing findings. Each work package will develop an impact plan which will include a timeline for communication activities and outputs.

The primary audience for SPECTRUM research will be policymakers at all tiers of government. However, impact will also be enhanced through communication with other research users: civil society, health organisations and practitioners, advocacy partners, research funders and the media, and diverse public audiences. Our communication will be tailored to each of these groups. Messages arising from the evidence generated through SPECTRUM research will be co-produced and incorporated into research outputs. Messages will focus on key SPECTRUM findings in relation to policy, practice and governance options to address commercial determinants of health within complex systems, effectiveness and economic analysis of policy and practice interventions and reduction of inequalities, and mental ill-health and stigma.

### Outputs

In addition to peer-reviewed publications, SPECTRUM will deliver a range of outputs. For example, we will develop new conceptual models on the commercial determinants of health (WP2) and on systems-level impacts of alcohol advertising and regulatory responses, and in a separate case study, to better understand on conflicts of interest (WP1). New analytic frameworks will also be developed, including modelling infrastructure to evaluate and appraise the impact of alcohol and tobacco policies across the wider economy and enable comparisons in these impacts to be drawn across England, Wales and Scotland (WP4). The conceptual models we develop will be presented as infographics to share with policy makers and NGOs to enhance understanding of the issues and applications of these models (i.e., WP2). We will also develop consensus principles/guidance for management of conflicts of interest in relationships between local/national policy or research stakeholders and commercial actors in the alcohol, tobacco/nicotine and food sectors (WP8), and new co-designed campaign messaging (to inform the work of Cancer Research UK (CRUK), PHE etc.) to avoid stigmatising vulnerable groups (WP7). We will also prepare infographics or short animations for the general public to raise critical consciousness of corporate influence on health and society (WP2) and illustrated reports of child and youth exposure to alcohol, tobacco and HFSS food promotion/normalisation through popular media (WP6). Open access websites (WP3) will report monthly survey results from the tobacco and alcohol toolkits for England, Scotland and Wales. A publicly accessible mapping platform (WP5) will allow a range of research users to access bespoke information on tobacco, alcohol and food environments (outlet density, pricing, harms) by postcode or other geographic levels (e.g. licensing board, local authority) across GB. These outputs will be synthesised in a range of policy options briefing papers and press releases that highlight the availability of infographics and illustrated reports will synthesise these outputs and present them in a format tailored for specific audiences. Finally, SPECTRUM will partner with the Obesity Health Alliance to produce an independent obesity strategy for the UK that includes ambitious objectives to be achieved in both the short and longer term. This will include policy recommendations and priorities for future research, which will inform our work and that of other researchers and research users.

### Covid-19 pandemic

The COVID-19 pandemic has caused a tragic loss of life and unprecedented change across the globe. The pandemic has resulted in social and emotional upheaval not seen since the Second World War. Markets and corporations have been as affected as any other sector, arguably even more so. The pandemic has changed business models and practices across multiple sectors. In some sectors, high volume, low cost business models are struggling to survive, for example in the service sector. Yet in other areas, such as the retail sector high volume, low cost businesses are booming. The pandemic illustrates that the commercial determinants of health are more important than ever for health. SPECTRUM will shine a light on these determinants of health and work with policy and practice partners during and following the COVID-19 crisis.

## Data availability

No data are associated with this article.
