# Supplementary material for: The SPECTRUM Consortium: a new UK Prevention Research Partnership consortium focussed on the commercial determinants of health, the prevention of non-communicable diseases, and the reduction of health inequalities
Source: Wellcome Open Res. 2021 Jan 14;6:6. [Version 1] doi: 10.12688/wellcomeopenres.16318.1 (PMC7931256; doi:10.12688/wellcomeopenres.16318.1)
Supplement: Peer review history

# Peer review: Reviewer response for version 1

**DOI:** 10.21956/wellcomeopenres.17937.r42602

**Published:** 2021-03-03

**Authors:** Andrew Wilson

**Affiliations:** 1 Menzies Centre for Health Policy, The University of Sydney, Sydney, NSW, Australia; 2 The Australian Prevention Partnership Centre, Sax Institute, Sydney, NSW, Australia

This Open letter describes the composition, aims, objectives, work plans and work processes and governance of SPECTRUM, a multi-institution partnership to address the negative contributions of some commercial determinants on health and wellbeing.

 The impact of marketing of products that increase the risk of many chronic diseases including the targeting of vulnerable populations has been well documented but interventional action, what works and what doesn't, has been much less systematically planned, implemented and evaluated. The history of progressive successful action in relation to one product, tobacco, provides an indication of what can be achieved, but there is a greater challenge when the products are less definable (eg. unhealthy foods) and/or more embedded (eg. alcohol).

 The Letter describes a comprehensive approach across 8 research streams that SPECTRUM will undertake to more systematically address countering commercially driven interests. Work program 1 "Using systems approaches to understanding determinants and address harms' is a key recognition that the world that it seeks to influence is complex, indeed particularly for food and nutrition. Work Program 4: "Economic and health impact synthesis to inform policy and practice decisions identifies mainly work in tobacco and alcohol but hopefully will progressively move into the more complex issues of the economics of unhealthy foods and some of the economic tools beyond taxation that may be relevant to that problem.

 SPECTRUM is a great start and hopefully can further expand its international linkages as many of commercial interests of concern are international and in the case of tobacco and alcohol are taking greater interests in emerging economy countries.

Does the article adequately reference differing views and opinions?

Yes

Are all factual statements correct, and are statements and arguments made adequately supported by citations?

Yes

Is the Open Letter written in accessible language?

Yes

Where applicable, are recommendations and next steps explained clearly for others to follow?

Not applicable

Is the rationale for the Open Letter provided in sufficient detail?

Yes

Reviewer Expertise:

health policy, health systems research, epidemiology

I confirm that I have read this submission and believe that I have an appropriate level of expertise to confirm that it is of an acceptable scientific standard.

# Peer review: Reviewer response for version 1

**DOI:** 10.21956/wellcomeopenres.17937.r42598

**Published:** 2021-02-15

**Authors:** Petra Meier

**Affiliations:** 1 University of Glasgow, Glasgow, UK

This open letter is a very accessible and well-written introduction to SPECTRUM, a multi-partner UK Prevention Research Partnership funded consortium that seeks to reduce health harm and health inequalities arising from the commercial determinants of health. Given the hefty burden of disease associated with the problematic consumption of alcohol, tobacco, fat, sugar and additives, this is an important public health policy area. 

 The proposed programme of work investigates this topic from different, often novel perspectives and uses methodological triangulation to create new insights. The work is guided by a strong theory of change, divided into meaningful work packages and valuable practice partner relationships. 

 Strong leadership will be required to ensure all the different elements optimally align, but the team is highly experienced in delivering such large-ranging programmes.

Does the article adequately reference differing views and opinions?

Yes

Are all factual statements correct, and are statements and arguments made adequately supported by citations?

Yes

Is the Open Letter written in accessible language?

Yes

Where applicable, are recommendations and next steps explained clearly for others to follow?

Yes

Is the rationale for the Open Letter provided in sufficient detail?

Yes

Reviewer Expertise:

Health policy,  health inequalities, alcohol policy, systems approaches

I confirm that I have read this submission and believe that I have an appropriate level of expertise to confirm that it is of an acceptable scientific standard.
